# The *Drosophila* FET ortholog Cabeza is an essential cofactor for ETV4-mediated activation of GGAA microsatellite neoenhancers

**DOI:** 10.1093/g3journal/jkag265

**Published:** 2026-09-22

**Authors:** Cristina Molnar, Jose Reina, Jaume Mora, Cayetano Gonzalez

**Affiliations:** Institute for Research in Biomedicine (IRB Barcelona), The Barcelona Institute of Science and Technology, Barcelona 08028, Spain; Institute for Research in Biomedicine (IRB Barcelona), The Barcelona Institute of Science and Technology, Barcelona 08028, Spain; Developmental Tumor Biology Laboratory, Institut de Recerca Sant Joan de Déu, Esplugues de Llobregat, Barcelona 08950, Spain; Pediatric Cancer Center Barcelona (PCCB), Hospital Sant Joan de Déu Barcelona, Esplugues de Llobregat, Barcelona 08950, Spain; Institute for Research in Biomedicine (IRB Barcelona), The Barcelona Institute of Science and Technology, Barcelona 08028, Spain; Institució Catalana de Recerca i Estudis Avançats (ICREA), Barcelona 08010, Spain

**Keywords:** ETS factors, GGAA microsatellites, ETV4, ERG, neoenhancer, EWS::FLI1, FET family, EWSR1

## Abstract

The conversion of transcriptionally silent GGAA microsatellites (GGAAµSats) into functional enhancers by FET::ETS oncogenic fusions is a hallmark of Ewing sarcoma. However, emerging evidence implicates nonfused, full-length oncogenic E26 transformation-specific (ETS) transcription factors in activating these repeats in other malignancies. Evaluating the *in vivo* transcriptional requirements of various human ETS factors in *Drosophila*, we found that human ETS variant transcription factor 4 (ETV4) associates with and robustly activates GGAAµSats in a tissue-specific manner. This activation is strongly inhibited by the human ETS repressor ETS variant transcription factor 6 (ETV6). Taking advantage of low genetic redundancy in *Drosophila*, we identified Cabeza (Caz), the single fly FUS/EWSR1/TAF15 family (FET) ortholog, as a necessary cofactor for ETV4-mediated transcription at GGAAµSats. Conversely, EWS::FLI1-mediated transcriptional activation of GGAAµSats is entirely independent of endogenous Caz, highlighting the distinct mechanics of covalent tethering vs noncovalent physical complexes. Collectively, our findings provide in vivo evidence that nonfused ETS factors cooperate with endogenous FET proteins to drive transcription from silent GGAA repeats, mechanistically validating this regulatory transformation known to operate as an oncogenic mechanism beyond Ewing sarcoma.

## Introduction

The conversion of transcriptionally silent GGAA microsatellites (GGAAµSats) into functional enhancers is widely recognized as a hallmark of Ewing sarcoma. This process is driven by oncogenes composed of the N-terminal domain of a FET family protein (fused in sarcoma [FUS], Ewing sarcoma RNA binding protein [EWSR1], or TATA-box binding protein associated factor [TAF15]) joined to the DNA-binding domain of an ETS factor such as Friend leukemia integration 1 (FLI1), ETS-related gene (ERG), and others. The most frequent of such oncogenes is EWS::FLI1, which is the driver for more than 80% of all Ewing sarcomas (Delattre et al. 1994; Sankar and Lessnick 2011; Crompton et al. 2014; Grünewald et al. 2018). The activation of GGAA microsatellites by these fusion proteins induces a tumor-specific gene regulatory program representing a critical mechanism in the pathogenesis of Ewing sarcoma (Gangwal et al. 2008; Guillon et al. 2009; Patel et al. 2012; Johnson et al. 2017; Boulay et al. 2018; Riggi et al. 2021).

ETS proteins play critical roles in normal tissue development and homeostasis and have been implicated as key drivers in a variety of human cancers. ETS proteins are characterized by a highly conserved 5′-GGA(A/T)-3′ DNA-binding motif (Sharrocks et al. 1997; Hsu et al. 2004; Hollenhorst et al. 2011; Cooper et al. 2015). Although many ETSs share the ability to bind GGAA-derived sequences, their capacity to induce significant transcriptional activity through these repeats varies significantly (Gangwal et al. 2010). The FET family proteins FUS, EWSR1, and TAF15 are characterized by intrinsically disordered, low-complexity prion-like domains (PrLDs). These domains facilitate multimerization, physiological liquid–liquid phase separation, and, under certain conditions, pathological protein aggregation (Thomsen et al. 2013; March et al. 2016; Ahmed et al. 2021).

In Ewing sarcoma, the multimerization of the EWS::FLI1 fusion protein via the EWSR1-derived N-terminal PrLD plays a pivotal role in recruiting the BAF complex to GGAAµSats (Boulay et al. 2017; Johnson et al. 2017). Similarly, the endogenous EWSR1 protein interacts with the BRG1 (SMARCA4) subunit of the BRG1/BRM-associated factor (BAF) complex through its own N-terminal PrLD—a process that is likewise essential for the oncogenic function of EWS::FLI1 (Boulay et al. 2017). This interaction is disrupted by tyrosine-to-serine mutations within the EWS::FLI1 PrLD, suggesting that these proteins colocalize and function through a mechanism of co-phase separation (Ahmed et al. 2021).

Despite their prominent relevance in Ewing sarcoma, emerging evidence suggests that nonfused, full-length ETS transcription factors can also engage and activate GGAAµSats in other malignancies. For instance, in prostate cancer, the oncogenic ETS proteins ERG, ETV1, ETV4, and ETV5 have been shown to activate GGAA repeat reporters (Kedage et al. 2016). These oncogenic ETS proteins are overexpressed as full-length or N-terminally truncated forms in approximately half of all cases (Clark et al. 2007; Kedage et al. 2016). Furthermore, in B-cell acute lymphoblastic leukemia (B-ALL), ERG directly binds to and transforms GGAAµSats into active enhancers, thereby sustaining the leukemic gene expression signature (Kodgule et al. 2023). Crucially, this regulatory transformation is gated by the status of the ETS repressor ETV6: it occurs only upon mutations that cause the loss of ETV6 or the inactivation of its DNA-binding domain (Bohlander 2005). Under normal conditions, ETV6 binds these GGAA repeats to antagonize the activating effects of ERG, serving as a molecular safeguard against leukemic reprograming (Kodgule et al. 2023).

Similar to FET::ETS fusion proteins, activation of GGAA microsatellites by full-length, aberrantly expressed ETS factors in prostate cancer is also suspected to require PrLD-dependent multimerization via FET family proteins (Kedage et al. 2016). However, in this context, in contrast to a chimeric fusion, multimerization is likely mediated via noncovalent protein–protein interactions between the full-length ETS factors and FET proteins. While current evidence strongly implicates EWSR1 in this process, definitive proof remains elusive because the ubiquitous expression and functional redundancy of FUS, EWSR1, and TAF15 complicate efforts to isolate their individual contributions within human cancer cells (Andersson et al. 2008).

We have previously shown that human EWS::FLI1 reproduces its neomorphic activation of transcription from synthetic GGAAµSat reporters in *Drosophila* (Molnar et al. 2022). Prompted by the evidence that full-length ETS factors can also activate these elements in human cancer cells, we aimed to explore the requirements for this activation using our *Drosophila* platform.

In this study, we systematically evaluate the transcriptional potential of various human oncogenic ETS factors at GGAA repeats and investigate the requirement for the single *Drosophila* FET ortholog, Caz. We show that ETV4 associates with and activates GGAA repeats in a length- and tissue-specific manner and demonstrate that the endogenous Caz protein is a critical determinant for converting these microsatellites into functional regulatory elements. We also show that human ETV6 functions as a strong inhibitor of this process in *Drosophila*. Moreover, by utilizing the TransTimer reporter system (He et al. 2019), we provide evidence of how human EWSR1 can substitute for its fly counterpart. Altogether, these findings provide substantial in vivo evidence of the functional conservation between human FET proteins and *Drosophila* Caz and confirm the essential role of endogenous FET protein in mediating the conversion of GGAAµSats into neoenhancers by nonfused oncogenic ETS transcription factors.

## Methods

### Fly stocks

The following fly stocks were used in this study: *antr-Gal4* (Lee et al. 2018), *sal^EPv^-Gal4* (Cruz et al. 2009), *hs-Gal4* (RRID:BDSC_1799), *longGMR-Gal4* (RRID:BDSC_8121), *UAS-mCD8-GFP* (RRID:BDSC_5130), *UAS-EWSR1* (RRID:BDSC_79592), *UAS-caz^RNAi^* (RRID:BDSC_32990 and RRID:BDSC_34839), and *UAS-mCherry^RNAi^* (RRID:BDSC_35785). The GGAAµSat reporter lines 20 × (GGAA)µSat > YFP, 10 × (GGAA)µSat > YFP, and 3 × (GGAA)µSat > YFP, as well as the *UAS-EWS::FLI1_FS_* line and *UAS-FLI1*, were previously described (Molnar et al. 2022). The following transgenic lines were generated and used in this work: *UAS-ERG*, *UAS-ETV1*, *UAS-ETV4*, *UAS-ETV6*, and *20* × *(GGAA)*µ*Sat > TransTimer*. All crosses, including controls, were maintained at 25 °C. FlyBase was used as a resource throughout this study (Öztürk-Çolak et al. 2024).

### Immunohistochemistry and microscopy

Immunostaining of salivary glands, wing discs, and accessory glands was performed as follows. Salivary glands and wing discs were dissected from wandering third instar larvae, and accessory glands from 4 to 5 day-old adult males, in phosphate-buffered saline (PBS). Tissues were fixed for 30 min in 4% formaldehyde with 0.3% TritonX-100, followed by 3 washes of 15 min each in PBS with 0.3% Triton X-100. DNA was stained with 4′,6-diamidino-2-phenylindole (DAPI). Salivary glands, wing discs, and accessory glands were mounted in Vectashield (Molecular Probes). Images were acquired with a Leica SP8 confocal microscope. The objective used was HC PL APO CS2 40×/1.30 OIL. Images were acquired at zoom 1. All immunofluorescence images shown correspond to a single optical section (Z-plane) and were processed using ImageJ.

### Quantification and statistical analysis

Fluorescence quantification in salivary glands was performed using ImageJ by calculating mean gray values in a single focal plane per gland from images acquired with a Leica SP8 confocal microscope. For the 3×, 10×, and 20× (GGAA)µSat > YFP reporter lines, mean gray values were measured for each region of interest per salivary gland (10 salivary glands per genotype). These data were analyzed using Welch's ANOVA followed by Dunnett's T3 multiple comparisons test.

For experiments in which multiple nuclei were quantified within each salivary gland, the salivary gland was considered the biological replicate and individual nuclei were treated as nested subsamples. In the ETV4/ETV6 experiment, destabilized GFP (dGFP) and RFP fluorescence was quantified in 5 nuclei from each of 8 salivary glands per genotype. In the ETV4/Caz/EWSR1 experiment, dGFP and RFP fluorescence and the corresponding RFP/dGFP ratio were quantified in 4 nuclei from each of 14 salivary glands per genotype. In the EWS::FLI1/Caz experiment, YFP fluorescence was quantified in 5 nuclei per salivary gland; 8, 10, and 10 salivary glands were analyzed for the *ctrl^RNA^*^i^, *caz*^32990^, and *caz*^34839^ conditions, respectively.

For these nested datasets, statistical significance was assessed using nested 1-way ANOVA followed by Tukey's multiple comparisons test. In the corresponding graphs, each point represents the mean of the nuclear measurements obtained from 1 salivary gland, and horizontal black lines indicate the mean across salivary glands for each genotype. Statistical analyses and *P*-value calculations were performed using GraphPad Prism 10 for macOS. Compact letter displays were used to summarize multiple-comparison results; groups not sharing a letter were considered significantly different at an adjusted *P* < 0.05.

### Cloning and transgenesis

Codon-optimized sequences for *Drosophila melanogaster* corresponding to the proteins ETV4 (P43268-1), ETV1(NP_001357485.1), and ERG (P11308-4) were synthetized by GeneWiz. ETV6 (P41212) ORF was synthetized by Twist Bioscience. All ORF was cloned in the *pUASt-attB* vector in *EcoRI*-*XbaI*. The 20× (GGAA)µSat > TransTimer reporter was made by inserting a PCR fragment containing the transcriptional timer (He et al. 2019) in the *p20x(GGAA)*µ*Sat-YFP* vector (Molnar et al. 2022) in *EcoRI-BglII*. The fragments were amplified from *pUAST-IVS-syn21-nlsGFP-PEST-2A-nlsRFP-p10* (DGRC Stock 1498). The primers used were: *UP-ATG*: 5′-AGGGAATTGGGAATTCCAAAATGCCCAAGAAGAAGC-3′; *UP-PEST*: 5′-GCTTCTGCTAGGATCAATGTG-3′; *DO-PEST*: 5′-CACATTGATCCTAGCAGAAGC-3′, and *DO-STOP*: 5′-CCGCGGCCGCAGATCTTTATTTTAAAAACGATTCATTCTA-3′.

All PCR amplifications were performed using KOD polymerase (Merck). Cloning was performed using In-Fusion Snap Assembly (Takara). Primers were ordered from Sigma.

Transgenic fly lines were generated by BestGene Inc. using the attP16 docking site for the 20× (GGAA)µSat > TransTimer transgene, the *M{3xP3-RFP.attP’}ZH-51C* docking site for the *UAS-ETV1*, *UAS-ERG*, and *UAS-ETV4* transgenes, and the *M{3xP3-RFP.attP}ZH-86Fb* docking site for the *UAS-ETV6* transgene. All transgenic lines used in this study were sequenced to confirm the presence and integrity of the correct transgene.

### Chromatin immunoprecipitation-quantitative PCR

Chromatin immunoprecipitation (ChIP) experiments were performed essentially as described (Climent-Cantó et al. 2020). A total of 300 larval salivary glands were dissected in 1× PBS and fixed with 1.8% formaldehyde for 10 min at room temperature. Samples were then washed in wash buffer A (10 mM HEPES pH 7.9, 10 mM EDTA, 0.5 mM EGTA, 0.25% Triton X-100), followed by wash buffer B (10 mM HEPES pH 7.9, 100 mM NaCl, 1 mM EDTA, 0.5 mM EGTA, 0.01% Triton X-100). Salivary glands were pelleted, resuspended in 900 µl of lysis buffer (10 mM Tris-HCl pH 8.0, 1 mM EDTA, 1% SDS, and 1 mM 4-(2-aminoethyl)benzenesulfonyl fluoride (AEBSF)), and sonicated using a Bioruptor Pico sonication device (Diagenode) for 9 cycles of 30 s ON and 30 s OFF to obtain chromatin fragments of 200 to 500 bp. After sonication, lysates were adjusted to 1% Triton X-100, 0.1% sodium deoxycholate (DOC), 140 mM NaCl, 1% SDS, and 1 mM AEBSF. Samples were centrifuged, and the supernatant was collected. For each immunoprecipitation, 1 ml of chromatin was used, and 40 μl were saved as input sample. Chromatin aliquots were precleared with 50 µl of Dynabeads Protein A (Thermo Fisher Scientific) for 3 h at 4 °C. Immunoprecipitations were performed using anti-ETV4 antibody (E1W1G, Cell Signaling Technology) or anti-DYKDDDDK Tag antibody as mock control (D6W5B, Cell Signaling Technology). A total of 4 µg of the corresponding antibody was added to each sample and incubated overnight at 4 °C on a rotating wheel. The following morning, 50 µl of BSA-blocked Dynabeads Protein A was added to each sample and incubated for 3 h at 4 °C. Beads were then collected on a magnetic rack and washed with radioimmunoprecipitation assay buffer buffer, followed by 1 wash with LiCl wash buffer (250 mM LiCl, 10 mM Tris-HCl pH 8.0, 1 mM EDTA, 0.5% NP-40, and 0.5% DOC) and 2 washes with TE buffer. Beads were treated with DNase-free RNase A/T1 (Thermo Fisher Scientific) for 30 min at 37 °C. Crosslinks were reversed by adjusting samples to 1% SDS, 0.1 M NaHCO_3_, and 0.2 mg/mL Proteinase K, followed by overnight incubation at 65 °C. DNA was purified using the QIAquick PCR Purification Kit (QIAGEN), according to the manufacturer's instructions.

Purified DNA from 2 independent ChIP experiments was analyzed by quantitative PCR (qPCR) using PowerUp SYBR Green Master Mix (Thermo Fisher Scientific). qPCR was performed using primers targeting the 20× (GGAA)µSat region and an SV40 region of the reporter construct as a control region. The primers used were: *20xGGAA-+1F*: 5′-CATGCCTGCAGGTGAGGGAG-3′; *20XGGAA-R*: 5′-CTCGCTAGAGTCTCCGTTTCTTTTCC-3′; *SV40-F*: 5′-TGCTGACTCTCAACATTCTACTC-3′; and *SV40-R*: 5′-CTCTAGTCAAGGCACTATACATCAA-3′. The qPCR results were analyzed using the fold change method, and ChIP enrichment was calculated relative to input DNA.

## Results

### ETV4 drives length-dependent transcription from GGAA microsatellites

To evaluate the capacity of human ETS transcription factors to activate GGAA microsatellites (GGAAµSats) in *Drosophila*, we expressed human ERG, ETV1, FLI1, and ETV4 in various *Drosophila* tissues, including the salivary glands, accessory glands, and wing and eye-antennal imaginal discs. These experiments were conducted in a strain harboring the 20× (GGAA)µSat > TransTimer reporter, in which dGFP reports recent transcriptional activity whereas the more stable RFP signal reflects cumulative transcriptional output over time. The UAS-ERG, UAS-ETV1, and UAS-ETV4 constructs were built in the same pUASt-attB vector backbone and integrated at the same docking site (ZH-51C), so they are subject to comparable position effects. *UAS-FLI1* was previously described (Molnar et al. 2022).

All 4 bring about a smaller than wild-type salivary glands, providing phenotypic evidence that they are expressed. However, while ERG, ETV1, and FLI1 failed to induce reporter expression in any examined tissue, ETV4 robustly activated the 20× (GGAA)µSat > TransTimer reporter in salivary and accessory glands, though not in imaginal or eye-antennal discs (Fig. 1a; Supplementary Fig. 1, a and b). Supporting this observation, ChIP-qPCR from larval salivary glands expressing ETV4 (*hs-Gal4 > UAS-ETV4*) demonstrated a specific enrichment of ETV4 at the 20× GGAA sequence (Fig. 1b). These findings demonstrate that ETV4 is a potent activator of GGAA microsatellites in an in vivo context in *Drosophila*. Moreover, this capacity is strictly governed by the cellular context, as among the tissues that we tested activation was restricted to glandular tissues and absent in imaginal discs. This suggests that ETV4 requires a permissive environment—potentially involving lineage-specific cofactors or distinct chromatin states—to initiate transcription from these repetitive elements. Moreover, salivary and accessory glands differ markedly from imaginal discs in ploidy, which could also contribute to this effect.

**Fig. 1.**
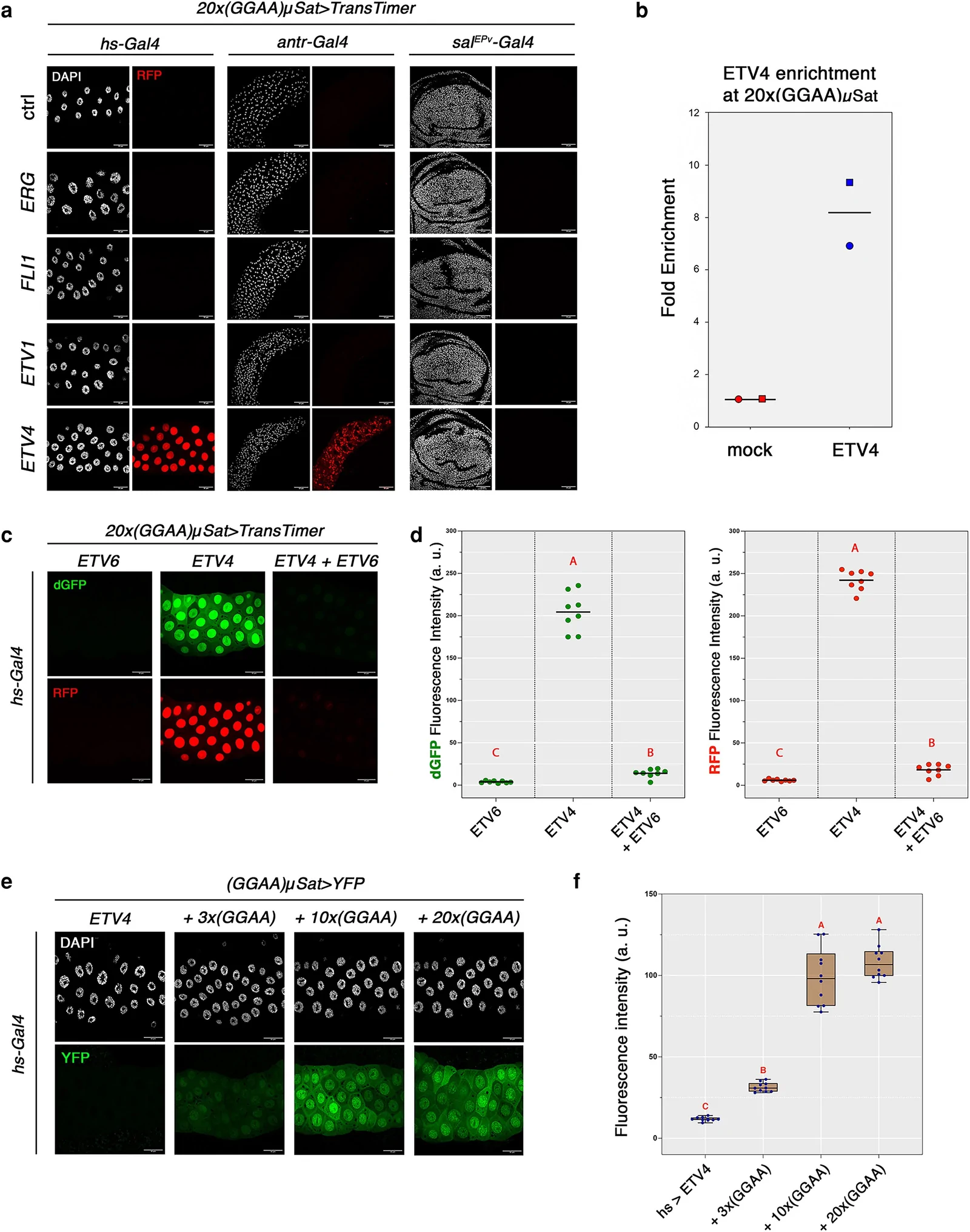
ETV4 drives tissue-specific and length-dependent transcription from GGAA microsatellites. a) Representative confocal images of salivary glands, accessory glands, and wing imaginal discs expressing human *ERG*, *ETV1*, *FLI1*, or *ETV4* under the control of *hs-Gal4*, *antr-Gal4*, or *sal^EPv^-Gal4*. DAPI stained nuclei and RFP reports transcriptional activation. Only ETV4 induces RFP expression from the 20× (GGAA)µSat reporter in salivary and accessory glands. Scale bar, 50 µm. b) ChIP-qPCR from larval salivary glands carrying the 20× (GGAA)µSat > TransTimer reporter and expressing ETV4 under the control of *hs-Gal4*, showing enrichment of the 20× (GGAA) sequence in chromatin immunoprecipitated with anti-ETV4 antibody relative to the anti-DYKDDDDK mock control. Individual values from 2 independent ChIP experiments are shown (*n* = 2; 150 pairs of salivary glands per experiment). c) Representative confocal images of salivary glands expressing ETV6 (*hs-Gal4 > UAS-ETV6*), ETV4 (*hs-Gal4 > UAS-ETV4*), or both (*hs-Gal4 > UAS-ETV4*; *UAS-ETV6*). dGFP and RFP report transcriptional activation. ETV6 strongly reduces dGFP and RFP fluorescence. Scale bar, 50 µm. d) Quantification of dGFP and RFP fluorescence intensity from glands shown in (c). Co-expression of ETV6 in ETV4-expressing glands strongly reduces both dGFP and RFP levels. Each point represents the mean fluorescence intensity of 5 nuclei from 1 salivary gland, and horizontal lines indicate the mean across salivary glands for each genotype. Statistical analysis was performed on nuclear measurements nested within salivary glands using nested 1-way ANOVA followed by Tukey's multiple comparisons test. Groups not sharing a letter are significantly different (adjusted *P* < 0.05). *n* = 8 salivary glands per genotype, with 5 nuclei quantified per gland. a.u., arbitrary units. e) Representative confocal images of larval salivary glands expressing ETV4 (*hs-Gal4 > UAS-ETV4*) and carrying (GGAA)µSat > YFP constructs with 3, 10, or 20 tandem GGAA repeats. DAPI staining marks nuclei and YFP reports transcriptional activation. The 3× (GGAA)µSat reporter shows weak activation, whereas 10× (GGAA)µSat and 20× (GGAA)µSat reporters display strong YFP signal. All images were acquired under identical confocal settings. Scale bar, 50 µm. f) Quantification of YFP fluorescence intensity in salivary glands for each reporter. ETV4-dependent activation was significantly lower from the 3× (GGAA)µSat reporter compared with the 10× and 20× reporters, indicating a repeat-length threshold for effective transcription. No significant difference was observed between the 10× and 20× reporters. Different letters indicate statistically significant differences between groups (Welch's ANOVA followed by Dunnett's T3 multiple comparisons test, adjusted *P* < 0.05). *n* = 10 salivary glands per each condition. a.u., arbitrary units.

We then tested the effect in *Drosophila* of the ETS transcriptional repressor ETV6. In human B-ALL cancer cells, activation of GGAA repeats by oncogenic ETS proteins and the consequent dysregulation of the corresponding transcriptome signature can only occur upon inactivation of ETV6 (Bohlander 2005; Kodgule et al. 2023). Likewise, we found that expression of human ETV6 strongly suppressed ETV4-dependent transcription activation from 20× (GGAA)µSat down to background levels in salivary glands (Fig. 1, c and d). This inhibitory effect of ETV6 further underscores the parallelism between ETV4-driven activation of GGAA repeats in *Drosophila* and human cells. It also reinforces the idea that GGAA repeat activity arises from a dynamic interplay between activating and repressive ETS factors (Lu et al. 2023).

In human cells, the number of GGAA repeats is critical for ETS-driven transcriptional activation (Kedage et al. 2016; Kodgule et al. 2023). To determine the relationship between repeat length and ETV4-mediated activation and to enable direct comparison with our previous EWS::FLI1 study, we quantified YFP fluorescence in salivary glands expressing ETV4 using the previously characterized 3×, 10×, and 20× (GGAA)µSat > YFP reporters (Molnar et al. 2022) (Fig. 1, e and f). Compared to control flies lacking the reporter, the 3× (GGAA)µSat > YFP construct exhibited a modest but significant 3-fold increase in fluorescence (adj. *P* = 7.81 × 10^−10^). In contrast, both the 10× and 20× reporters showed a robust, 10-fold increase (adj. *P* = 4.35 × 10^−7^ and adj. *P* = 1.54 × 10^−9^, respectively). Notably, the levels of YFP upregulation elicited by ETV4 did not differ significantly between the 10× and 20× reporters (adj. *P* = 0.6547), suggesting a potential saturation of transcriptional activation at 10 GGAA repeats. These results demonstrate that transcriptional output correlates with GGAA repeat length, reaching a plateau at 10 repeats, consistent with cooperative binding models for tandem motifs (Guillon et al. 2009). Notably, this trend mirrors the EWS::FLI1-mediated activation of GGAA microsatellites observed in *Drosophila*, though the fusion oncogene requires a longer tract—20 repeats—to reach saturation (Molnar et al. 2022).

### The single *Drosophila* FET ortholog cabeza regulates ETV4-mediated GGAA-microsatellite transcription

In prostate cancer cells, EWSR1 is hypothesized to be an essential cofactor for oncogenic ETS factors to activate transcription from GGAA microsatellites (Kedage et al. 2016). However, evidence remains inconclusive because the effect of EWSR1 depletion is only partial, probably owed to the redundant functions of all 3 FET family members—FUS, EWSR1, and TAF15—which are ubiquitously expressed (Andersson et al. 2008).

Taking advantage of the fact that *Drosophila* possesses a single FET ortholog, Cabeza (Caz), we investigated whether Caz is required for ETV4-mediated transcriptional activity. To this end, we quantified the effect of Caz depletion on ETV4-driven transcriptional activation from the 20× (GGAA)µSat > TransTimer reporter (Fig. 2, a–d).

**Fig. 2.**
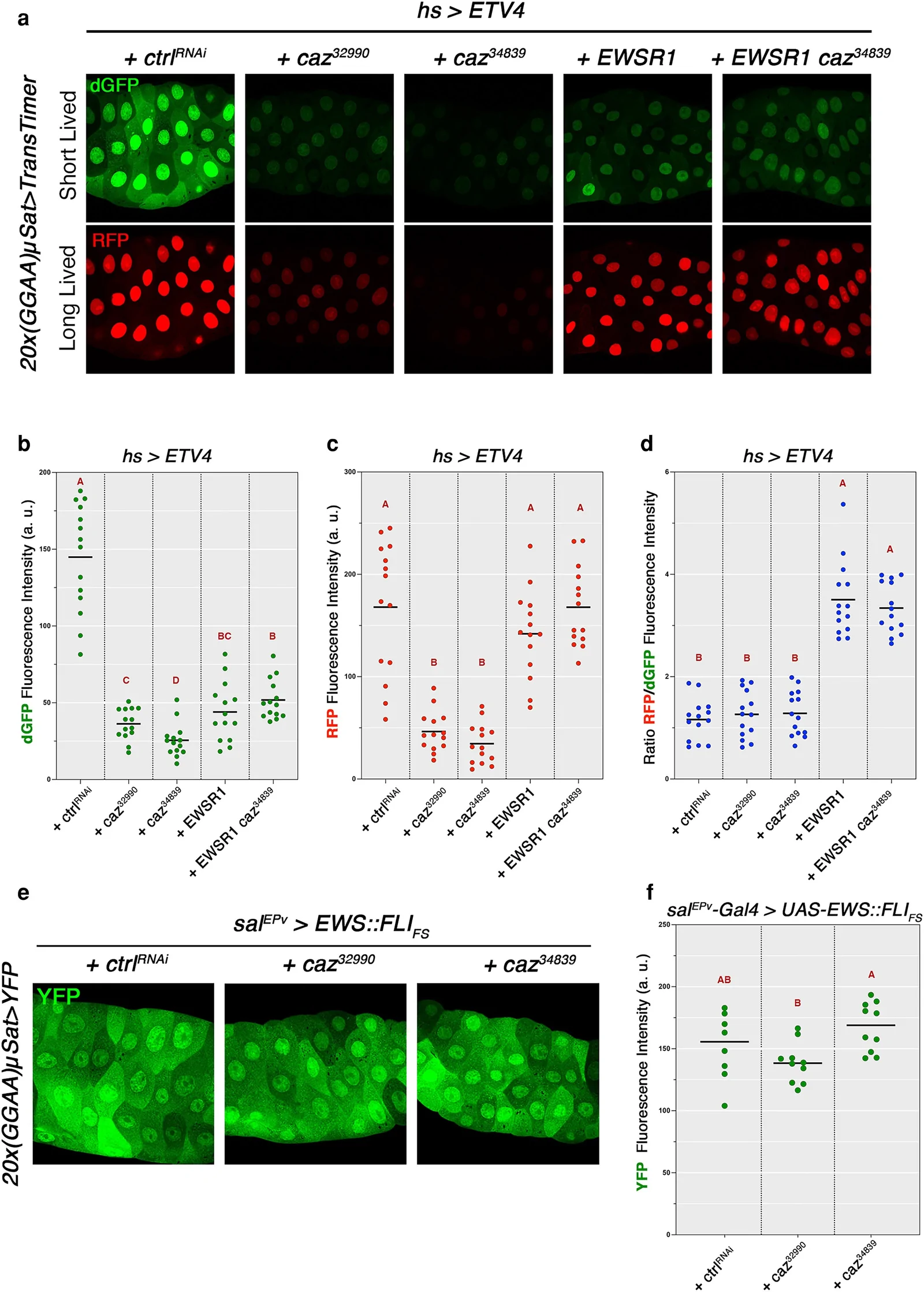
The *Drosophila* EWSR1 ortholog Cabeza is required for ETV4 to activate transcription from 20× (GGAA)µSat. a) Representative confocal images of salivary glands expressing *hs-Gal4 > UAS-ETV4* together with 20× (GGAA)µSat > TransTimer and either a control RNAi line (*ctrl^RNAi^ = mCherry^35785^-RNAi*), 2 independent *caz* RNAi lines (*caz^32990^* or *caz^34839^*), human EWSR1, or human EWSR1 together with *caz^34839^*. Depletion of *caz* with either RNAi line strongly reduced both short-lived dGFP and long-lived RFP fluorescence. Co-expression of EWSR1 in *caz*-depleted salivary glands (*EWSR1 caz^34839^*) partially restored reporter activity, with a stronger recovery of the RFP signal. b–d) Quantification of dGFP fluorescence intensity (b), RFP fluorescence intensity (c), and RFP/dGFP ratio (d) from glands shown in (a). Each point represents the mean of measurements from 4 nuclei within 1 salivary gland, and horizontal black lines indicate the mean across salivary glands for each genotype. Both *caz* RNAi lines significantly reduced dGFP and RFP fluorescence. Co-expression of EWSR1 in *caz*-depleted background restored RFP levels to values comparable to those observed with EWSR1 expression alone. Depletion of *caz* did not significantly alter the RFP/dGFP ratio compared to the control, whereas co-expression of EWSR1 either alone or in the *caz*-depleted background, significantly increases this ratio. *n* = 14 salivary glands per genotype, with 4 nuclei quantified per gland. e) Representative confocal images of salivary glands expressing EWS::FLI1 under the control of *sal^EPv^-Gal4*, which also drives expression in larval salivary glands, together with the 20× (GGAA)µSat > YFP reporter either *ctrl^RNAi^*, *caz^32990^*, or *caz^34839^*. f) Quantification of YFP fluorescence intensity from the genotypes shown in (e). Each point represents the mean fluorescence intensity of 5 nuclei from 1 salivary gland, and horizontal black lines indicate the mean across salivary glands for each genotype. Depletion of *caz* did not cause a consistent reduction in EWS::FLI1-driven reporter activity. *n* = 8, 10, and 10 salivary glands for *ctrl^RNAi^*, *caz^32990^*, and *caz^34839^*, respectively, with 5 nuclei quantified per gland. For (b–d) and (f), statistical analysis was performed on nuclear measurements nested within salivary glands using nested 1-way ANOVA followed by Tukey's multiple comparisons test. Groups not sharing a letter are significantly different (adjusted *P* < 0.05). a.u., arbitrary units for fluorescence intensity measurements.

Two independent *caz-*RNAi lines, *caz^32990^* and *caz^34839^*, with 50% and 80% reported knockdown efficiencies (Frickenhaus et al. 2015), were used in this study. Compared to a control RNAi (*mCherry^35785^*), expression of *caz^32990^* or *caz^34839^* in ETV4-expressing salivary glands resulted in a robust reduction of both the short-lived dGFP (adj. *P* = 1.46 × 10^−12^ and adj. *P* = 1.46 × 10^−12^, respectively) and the long-lived RFP fluorescence (adj. *P* = 5.73 × 10^−10^ and adj. *P* = 2.64 × 10^−11^, respectively) (Fig. 2, a–d). These strong phenotypes caused by 2 *caz-*RNAi lines that deplete Caz only partially strongly suggest that Caz is an essential cofactor, necessary for ETV4-mediated transcriptional activation at GGAAµSats in the *Drosophila* salivary gland.

To determine if the role of Caz in this context is functionally equivalent to human EWSR1, we quantified the effect of expressing human *EWSR1* on ETV4-driven transcriptional activation (Fig. 2, a–d). We found a significant difference between the short (dGFP) and the long-lived (RFP) reporters. In Caz wild-type salivary glands, *EWSR1* expression had no significant effect on the expression of the long-lived RFP signal, but significantly reduced the expression of dGFP fluorescence compared to the control RNAi (adj. *P* = 0.4546 and adj. *P* = 1.48 × 10^−12^, respectively). However, more importantly, *EWSR1* expression in Caz-depleted cells (*caz^34839^-RNAi*) fully rescued both the RFP and dGFP signals, back to the levels observed in Caz wild-type salivary glands expressing EWSR1 (Fig. 2, a–d). These results demonstrate EWSR1 and Caz functional homology in the activation of transcription from 20× (GGAA)µSat by ETV4 in *Drosophila* salivary glands. In addition, the complete restoration of the cumulative RFP signal indicates that EWSR1 is fully capable of sustaining total transcriptional output over time. Nonetheless, the partial rescue of the short-lived dGFP signal suggests that while EWSR1 supports the initiation of new transcription, it may possess lower kinetic efficiency than the native Caz within the *Drosophila* cellular environment.

Interestingly, ERG, ETV1, and FLI1, which failed to activate transcription from the GGAAµSat reporter when expressed on their own, remained silent upon co-expression with EWSR1 in salivary glands, accessory glands, or wing discs (Supplementary Fig. 2). This demonstrates that, like the endogenous Caz, human EWSR1 is not sufficient to confer transcriptional activity to any of these ETS proteins at GGAA repeats.

Mechanistically, our results are consistent with the proposal that the interaction between full-length EWSR1 and ETS proteins in human cancer cells mimics the activity of the corresponding oncogenic fusion which harbors its own PrLD and DNA-binding domain. Consistent with this hypothesis, we found that YFP levels in Caz-depleted cells in EWS::FLI1 expressing salivary glands remained indistinguishable from the control condition (adj. *P* = 0.4021 and adj. *P* = 0.2627 for *caz^32990^* and *caz^34839^*, respectively) (Fig. 2, e and f). This result indicates that the fusion oncogenic protein does not require Caz for GGAAµSat activation. Moreover, it also serves as an internal control showing that Caz depletion does not compromise transcription, reporter function, or Gal4-driven expression in general.

## Discussion

While the conversion of transcriptionally silent GGAA microsatellites (GGAAμSats) into functional enhancers is widely recognized as a hallmark driving the pathogenesis of Ewing sarcoma via FET::ETS fusion proteins like EWS::FLI1, this regulatory mechanism is also relevant across other cancer types through nonfused, full-length ETS transcription factors. In prostate cancer, overexpressed oncogenic ETS proteins such as ERG, ETV1, ETV4, and ETV5 actively engage and activate these GGAA repeat regions (Kedage et al. 2016). Similarly, in B-ALL, ERG transforms these microsatellites into active enhancers to sustain leukemic gene expression following the loss or inactivation of the ETS repressor ETV6 (Kodgule et al. 2023).

Using transgenic *Drosophila* lines carrying fluorescent reporters of GGAAμSat transcriptional activation, we have found that human ETV4 robustly activates transcription from GGAA microsatellites in vivo in the salivary and accessory glands, but not in other tissues like the wing and eye-antennal imaginal discs. This context-dependent activity suggests that tissue-specific factors may be required for ETV4 to engage and unlock these silent genomic elements. In contrast, GGAAμSat appears to remain transcriptionally silent upon expression of ERG, ETV1, and FLI1 in all 4 tissues examined. This result may reflect functional divergence among ETS factors with regard to their effect on GGAA repeats. However, a negative result cannot be taken as definitive proof that these factors cannot activate transcription from GGAA microsatellites in these tissues. Our study identifies Caz, the *Drosophila* ortholog of the human FET proteins as a critical requirement for ETV4 to activate transcription from GGAAμSats. Moreover, it also shows that human EWSR1 can partially substitute and compensate for the loss of Caz in the activation of transcription from 20× GGAAμSats by ETV4. These results demonstrate functional conservation between the fly and its human ortholog. Nonetheless, the observations derived from the GGAAμSat TransTimer reporter revealed a subtle difference in the kinetic profile for this rescue: human EWSR1 fully restores the cumulative transcriptional pool over time but only partially restores the “fresh” nascent signal compared to endogenous Caz. This suggests that while the human protein is functionally compatible, it may be less kinetically efficient within the *Drosophila* cellular environment than the native Caz ortholog.

Importantly, our finding that ERG, ETV1, and FLI1 remain unable to erase transcriptional silencing at GGAAμSats even when co-expressed with human EWSR1 further underpins the view that neither EWSR1 nor Caz is sufficient to confer microsatellite activation potential to all full-length ETS factors in vivo. This highlights the critical importance of additional lineage-specific cofactors, protein–protein interactions, or distinct differences in the baseline chromatin accessibility landscapes between mammalian and insect systems.

In prostate cancer, oncogenic ETS-dependent transcriptional activation of GGAAμSats has been suggested to be mediated through interaction with EWSR1 (Kedage et al. 2016). This observation led to propose that whether covalently tethered in a fusion protein or brought together through protein–protein interaction, the conjunction of an ETS DNA-binding domain and a FET protein prion-like low-complexity domain can erase transcription silencing at GGAAμSats. Our results fully substantiate this hypothesis.

However, while sharing foundational similarities, the transactivation mechanisms driven by covalent tethering vs physical interaction exhibit 3 distinct differences. First, while full-length FLI1 has no effect, the EWS::FLI1 fusion protein drives strong Caz-independent GGAAμSats transcriptional activation. Second, our data reveal that the transcriptional output of the full-length ETV4/Caz cooperative complex plateaus at 10 repeats. In contrast, the EWS::FLI1 fusion oncogene requires a 20-repeat saturation point in *Drosophila* (Molnar et al. 2022). This divergent length-dependence likely reflects differential cooperative binding of transcription factors and cofactors across multiple tandem motifs, which ultimately shapes chromatin accessibility and establishes distinct thresholds for effective enhancer output. Finally, the mechanisms driven by covalent tethering and physical interaction also differ regarding ETV6-mediated repression: in *Drosophila* salivary glands, ETV6 efficiently represses the activity of full-length ETV4 but has no effect on EWS::FLI1.

Taken together, our findings support a model in which full-length ETS transcription factors can activate transcription at GGAAμSats through interaction with FET family proteins. They also underscore the potential relevance of GGAAμSat-driven unscheduled transcription in other tumor types beyond Ewing sarcoma.

## Supplementary Material

jkag265_Supplementary_Data

## Data Availability

Strains and plasmids are available upon request. The authors affirm that all data necessary for confirming the conclusions of the article are present within the article and figures. Supplemental material available at G3 online.
