# Supplementary material for: The Drosophila FET ortholog Cabeza is an essential cofactor for ETV4-mediated activation of GGAA microsatellite neoenhancers
Source: G3 (Bethesda). 2026 Sep 22;16(11):jkag265. doi: 10.1093/g3journal/jkag265 (PMC13732429; doi:10.1093/g3journal/jkag265)
Supplement: Supplementary file 1 [file jkag265_supplementary_data.zip › Supplemental_Material_G3-2026-407218.docx]

**Supplementary Figure 1. ETS transgene phenotypes in salivary glands and tissue-specific activation of the GGAAµSat reporter by ETV4.**

**(a)** Representative confocal images of larval salivary glands expressing *ERG*, *FLI1*, *ETV1* or *ETV4* under the control of *hs-Gal4.* Only ETV4 induces YFP expression from the 20x(GGAA)µSat reporter. Expression of each ETS factor results in smaller salivary glands than in the control.

**(b)** Eye imaginal discs from larvae expressing mCD8-GFP or ETV4 in cells posterior to the morphogenetic furrow with *longGMR-Gal4*. ETV4 expression has no effect in the ommatidia development.

**Supplementary Figure 1 Alt text:** Salivary glands from larvae expressing ERG, FLI1, ETV1 or ETV4, showing smaller glands for all four ETS factors and yellow fluorescent protein reporter signal only with ETV4. Two eye imaginal discs from larvae expressing membrane targeted green fluorescent protein or ETV4 behind the morphogenetic furrow, showing similar regular ommatidial patterning in both genotypes.

**Supplementary Figure 2. Human EWSR1 has no effect on ETS factors to activate expression from 20x(GGAA)µSat in *Drosophila*.**

Representative confocal images of salivary glands **(a)**, accessory glands **(b)** and wing imaginal discs **(c)** co-expressing human ERG, ETV1, FLI1, or ETV4 with EWSR1 under the control of *hs-Gal4*, *antr-Gal4* or *sal^EPv^-Gal4*. DAPI (grey) stained nuclei and RFP (red) reports transcriptional activation. Only co-expression of EWSR1 and ETV4 induces RFP expression from the 20x(GGAA)µSat reporter in salivary and accessory glands. Scale bar, 50 µm.

**Supplementary Figure 2 Alt text:** Micrographs in three panels. a, salivary glands; b, accessory glands; c, wing imaginal discs, each expressing human EWSR1 together with ERG, ETV1, FLI1 or ETV4. Nuclei are grey. Red reporter signal appears only with EWSR1 and ETV4 in salivary and accessory glands, and is absent in wing discs.
